# Patterns of Cross-Continental Variation in Tree Seed Mass in the Canadian Boreal Forest

**DOI:** 10.1371/journal.pone.0061060

**Published:** 2013-04-11

**Authors:** Jushan Liu, Yuguang Bai, Eric G. Lamb, Dale Simpson, Guofang Liu, Yongsheng Wei, Deli Wang, Daniel W. McKenney, Pia Papadopol

**Affiliations:** 1 Department of Plant Sciences, University of Saskatchewan, Saskatoon, Canada; 2 Institute of Grassland Science, Northeast Normal University, and Key Laboratory of Vegetation Ecology, Ministry of Education, Changchun, P.R. China; 3 Natural Resources Canada, Atlantic Forestry Centre, Fredericton, Canada; 4 State Key Laboratory of Vegetation and Environmental Change, Institute of Botany, Chinese Academy of Sciences, Beijing, P.R. China; 5 College of Life Sciences, Northwest Agriculture & Forestry University, Yangling, P.R. China; 6 Natural Resources Canada, Great Lakes Forestry Centre, Sault Ste. Marie, Canada; DOE Pacific Northwest National Laboratory, United States of America

## Abstract

Seed mass is an adaptive trait affecting species distribution, population dynamics and community structure. In widely distributed species, variation in seed mass may reflect both genetic adaptation to local environments and adaptive phenotypic plasticity. Acknowledging the difficulty in separating these two aspects, we examined the causal relationships determining seed mass variation to better understand adaptability and/or plasticity of selected tree species to spatial/climatic variation. A total of 504, 481 and 454 seed collections of black spruce (*Picea mariana* (Mill.) B.S.P.), white spruce (*Picea glauca* (Moench) Voss) and jack pine (*Pinus banksiana* Lamb) across the Canadian Boreal Forest, respectively, were selected. Correlation analyses were used to determine how seed mass vary with latitude, longitude, and altitude. Structural Equation Modeling was used to examine how geographic and climatic variables influence seed mass. Climatic factors explained a large portion of the variation in seed mass (34, 14 and 29%, for black spruce, white spruce and jack pine, respectively), indicating species-specific adaptation to long term climate conditions. Higher annual mean temperature and winter precipitation caused greater seed mass in black spruce, but annual precipitation was the controlling factor for white spruce. The combination of factors such as growing season temperature and evapotranspiration, temperature seasonality and annual precipitation together determined seed mass of jack pine. Overall, sites with higher winter temperatures were correlated with larger seeds. Thus, long-term climatic conditions, at least in part, determined spatial variation in seed mass. Black spruce and Jack pine, species with relatively more specific habitat requirements and less plasticity, had more variation in seed mass explained by climate than did the more plastic species white spruce. As traits such as seed mass are related to seedling growth and survival, they potentially influence forest species composition in a changing climate and should be included in future modeling of vegetation shifts.

## Introduction

Seed mass or size is a key adaptive trait in plant life history and has important implications for species distribution, population dynamics and community structure [1], [2]. Changes in such traits as affected by global climate change will affect other life history traits of plants [3]. Within a species, seed size is a relatively stable trait compared to other plant parts; plants change the size of other parts first before changing seed size in response to changes in environmental conditions [4]. On the other hand, flexibility or plasticity in seed size is also an important trait, as it enables plants to adapt to heterogeneous and/or changing environments [5]. Variation in seed mass can be especially expected for widely distributed species because of the diversity in habitats and climate in widely distributed species. However, partitioning such variation according to genetic adaptation and adaptive phenotypic plasticity using the common garden approach is difficult, particularly for long-lived tree species.

Seed mass changes in response to environmental stresses and disturbances, and is presumed to be shaped by compromises between conflicting selective pressures [6], [7]. Seed mass is related to traits such as plant size, dispersal mode and plant life-span [8]. Large seeds have higher germination probability, greater seedling survivorship and higher competitive ability [7], perform better than small seeds under water, light and nutrient limitations [6], [9], and are more resistant to diseases [10]. Large seeds, however, can be more vulnerable to predation and have less dispersal potential by wind [11]. Moreover, resource constraints during seed provisioning can cause trade-offs between seed number and size, consequently leading to seed mass variation within and among plant species [12].

Environment conditions affect intra-specific variation in seed mass [8], [13]. Seed mass within a species usually decreases with increasing latitude [14], [15], [16], [17], partly due to decreasing solar radiation and photosynthate availability associated with decreases in temperature and growing season length from low to high latitude [16], [18]. Greater seed mass at low latitudes can be explained by the need of large seeds to produce seedlings with sufficient resources to cope with shaded understory conditions [14], [17], and the high metabolic costs of plant growth and maintenance at high temperatures [16]. Along a longitudinal gradient across Australia, seed mass increases with increasing aridity [15]. However, seed mass of annual species has been shown to decrease with increasing aridity at the community level [19]). Large seed size is an adaptation to shaded environment because seedlings with large seed reserves are more competitive than those from small ones [5], [13], [15]. Seed mass generally decreases with altitude, although it is inconsistent within species [15], [20]. However, correlation or partial correlation analyses were widely used in previous studies, but they cannot demonstrate causal effects.

Climate related reproductive adaptations can restrict species distributions, and thus are relevant to the prediction of tree population response to climate change [21]. Short term variation in weather conditions at the reproductive stage can influence the periodicity of seed production [22], and the quantity and quality of seeds [21], [23]. Warm and sunny weathers during flowering and seed development favour the production of large seeds [24]. However, high rainfall may lead to low success of pollination and fertilization and low seed quality [25]. Previous analyses of forest species distribution and potential shifts under climate change have focused mainly on growth, but understanding the reproductive responses of trees to climatic variation will shed light on the physiological mechanisms limiting the distribution and shaping adaptations of these species [26].

The cross-continental distribution of the Canadian Boreal Forest and its broad range of climate provide an excellent opportunity to study factors influencing spatial and temporal variation in tree seed mass. Climate varies from dry continental in the interior to wet maritime in coasts along with changes in disturbance regimes and disturbance–climate interactions, such as fire frequency, insect and pathogen attacks, and interspecific competition within the boreal forest. Black spruce (*Picea mariana* (Mill.) B.S.P.), white spruce (*Picea glauca* (Moench) Voss) and jack pine (*Pinus banksiana* Lamb) are among the dominant tree species with transcontinental distribution [27], [28], [29]. Black spruce is stress-tolerant and moderately fire-dependent, able to grow in cold permafrost soils and most common in wet soils and bogs [30], [31]. White spruce is a plastic species found in a wide variety of habitats and soil conditions and well adapted to the cold soils of permafrost zones [31]. Jack pine can be a pioneer species, is drought-resistant, deep snow-intolerant, and typically found in poor and sandy soils [21], [31].

Large-scale analyses on the correlations between plant functional traits (e.g. seed mass, plant height or leaf anatomy) and long term climatic variables have been used to determine geographic variation in such traits and plant adaptation to climate [8], [32], [33]. Because of the large samples size in such studies, plant traits are usually obtained from database or published literature covering various years. In this study, we examined the spatial patterns in seed mass of these species along gradients of latitude, longitude and altitude in the Canadian Boreal Forest, and attempted to identify relationships among long term climate regulating seed mass using Structural Equation Modeling (SEM). The following hypotheses were tested: 1) seed mass decreases with increasing latitude and altitude, and decreases from coasts to the interior, 2) temperature controls latitudinal and altitudinal variation of seed mass, and precipitation controls longitudinal variation, and 3) species-specific adaptation in seed mass to climate can be related to the ecological characteristics of these species.

## Materials and Methods

### Seed data

**Figure 1:**
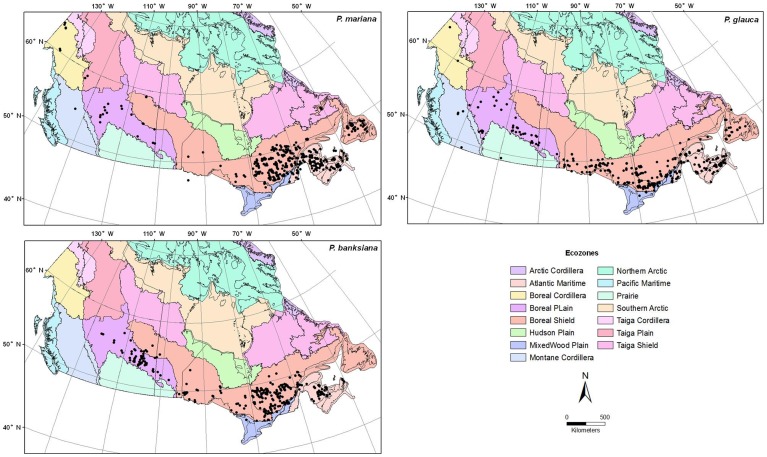
Location of seed collections of black spruce, white spruce and jack pine.

Seed data were obtained from the seed inventory database for black spruce, white spruce and jack pine across the Canadian Boreal Forest at the National Forest Genetics Research Centre, Canadian Forest Service (Fig. 1). The year of collection, provenance, collection type, latitude, longitude, altitude, and TSW (thousand- seed-weight) were extracted from the database. Collections with missing information such as latitude, longitude or seed mass were eliminated. The seed mass of multiple collections from the same site in the same year was averaged. A total of 504, 481 and 454 seed collections of black spruce, white spruce and jack pine, respectively, were used for this study, covering 377, 334 and 306 provenances. Mean seed mass was 1.26±0.24, 2.33±0.35 and 3.48±0.45 mg (mean ± SE) for black spruce, white spruce and jack pine, respectively.

### Climatic data

Ninety six baseline climate parameters (1971–2000) across North America were obtained from the Landscape Analyses and Application Section (LAAS), Great Lakes Forestry Centre (GLFC), Sault Ste. Marie, Ontario (Table S1). Bioclimatic parameters were generated with BIOCLIM (a Bioclimate Prediction System) and a digital elevation model (DEM) with the grid size of 10 km. Data for monthly solar radiation were generated using the Canadian Regional Climate Model (CRCM 4.2) over the North-American domain with a 45 km horizontal grid-size mesh (true at 60 °N) (http://www.cccma.ec.gc.ca/data/crcm423/crcm423.shtml). The climatic profiles for the three species included 96 variables (Table S1). McKenney et al. (2006) provided detailed definitions of the standard bioclimatic variables used [34].

### Statistical analysis

Simple correlation analyses were used to examine the relationship between seed mass and latitude, longitude and altitude for black spruce, white spruce and jack pine separately. To eliminate compounding effects, partial correlation analyses were performed between seed mass and each geographical variable by controlling the other two spatial variables [15]. Relationships between seed mass and climatic variables were also examined using simple correlation analysis. The geographical variation of seed mass was further assessed within two contrasting ecozones with the largest numbers of collections, the Atlantic Maritime and Boreal Shield ecozones.

Structural Equation Modeling (SEM) was used to quantify the networks of interactions among geographic and climatic variables hypothesized to control seed mass [35], [36]. SEM is a powerful tool for examining relationships among inter-correlated variables where the covariance structure indicated by a theoretical causal relationship (initial model) is tested against the observed variance-covariance matrix. A good fit between the theoretical and empirical covariance structures is a powerful confirmatory test of the theory used to develop the initial model [35]. In an SEM model each single-headed arrow represents a causal relationship where the variable at the tail of the arrow is a direct cause to the variable at the head, and a double-headed arrow indicates an unresolved correlation between two variables. An initial SEM was specified based on prior theoretical knowledge, and a χ^2^ test was used to determine whether the covariance structures implied by the model adequately fit the actual covariance structures of data. A non-significant χ^2^ test result (*P*>0.05) indicates adequate model fit.

Climate variables were screened to identify representative variables to be used in the SEM. Within each group (e.g. temperature, precipitation, evaporation and radiation), variables that measured annual means or totals and seasonal variability (if applicable) were first selected if they were significantly correlated with seed mass (*P*≤0.05, |*r*|≥0.20) (Table S1). Variables for specific periods were selected if annual means or totals did not meet the above criteria. The criteria were relaxed in the case of precipitation for jack pine and radiation for white spruce because of the overall low correlations of these variables with seed mass. Variables that were highly correlated with any of the selected variables were excluded (*P*≤0.05, |r|≥0.5). The resulting variables used in the SEM included annual mean temperature, temperature seasonality, total annual precipitation, precipitation seasonality, annual mean radiation, and fall evaporation for black spruce, temperature seasonality, total annual precipitation, growing season mean radiation, and growing season evaporation for white spruce, and growing season mean temperature, temperature range, annual total precipitation, annual mean radiation and growing season evaporation for jack pine. Because annual mean temperature and precipitation seasonality were included in SEMs for black spruce and jack pine, they added back to the model for white spruce (Fig. S1). These two variables did not affect the model fit of the white spruce model, and thus were included in the model.

Given data pre-screening in the development of initial models, these SEMs should be considered exploratory rather than confirmatory [35]. Observed variables rather than latent variables were used because these climatic variables are also commonly used in forest modeling [35]. Variables were standardized to similar ranges prior to analysis (latitude, longitude and temperature were divided by 10, altitude by 100, and precipitation by 1000) [36]. Scaling the variables to similar ranges is an important step to ensure model convergence. It was assumed in the initial models that seed mass is the result of climatic variables and spatial variables affect seed mass through climatic variables (Fig. S1). Bidirectional paths between latitude, longitude and altitude were included because of the inherited correlations among them due to the distribution range of these species. The χ^2^ test was used to determine whether the fit between model and data was adequate (*P*>0.05), and non-significant paths were retained in the final models [35], [36]. The analysis was conducted using the SEM package in the R 2.14.0 [37], [38]. The initial models did not have an adequate fits (black spruce: χ^2^_18_ = 1978.9, *P*<0.0001; white spruce: χ^2^_18_ = 1311.8, *P*<0.0001; Jack pine: χ^2^_13_ = 1340, *P*<0.0001).

**Figure 2:**
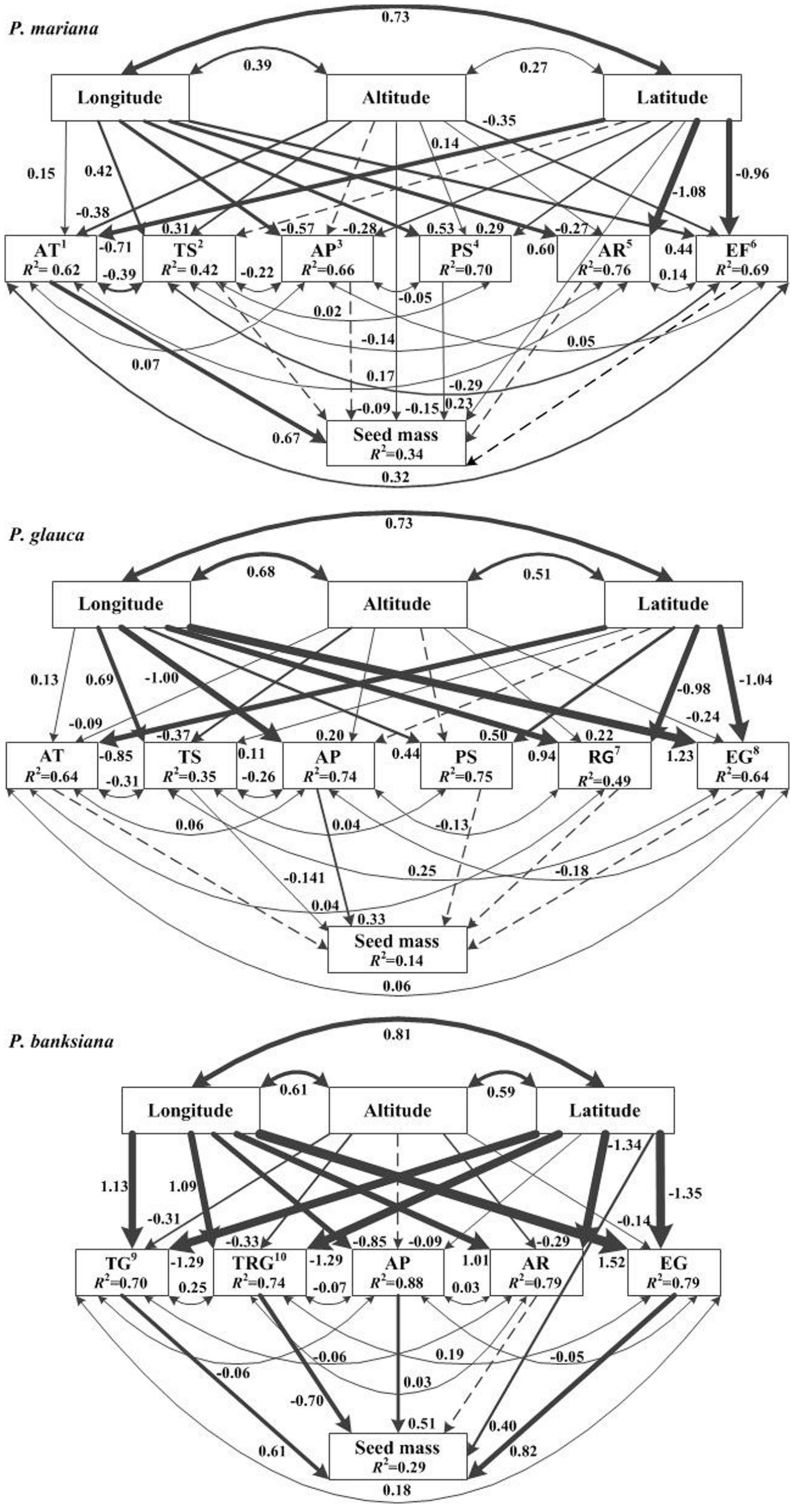
SEM for geographic variation of seed mass for black spruce, white spruce and jack pine. Non-significant paths are indicated by dotted arrows. The thickness of the solid arrows reflects the magnitude of the standardized SEM coefficients. Standardized coefficients are listed beside each significant path, as is the variance explained (r^2^) for each endogenous variable. ^1^AT, Annual temperature (°C); ^2^TS, Temperature seasonality (%); ^3^AP, Annual precipitation (mm); ^4^PS, Precipitation seasonality (°C); ^5^AR, Annual radiation (mrem); ^6^EF, Total potential evaporation in the fall (August to November) (mm); ^7^RG, Mean radiation in growing season (April to September) (mrem); ^8^EG, Total potential evaporation of growing season (April to September) (mm); ^9^TG, Mean temperature of growing season (April to September) (°C); ^10^TRG, Temperature range of growing season (°C).

Each model was modified according to modification indices until adequate model fit was achieved (*P*>0.05). Modification indices are a standard method for improving SEM model fit [35], [36]. Briefly, the user is provided with a list of how the addition of every possible direct or bi-directional relationship would improve model fit (reduce χ^2^ values). The modeler must select paths that are biologically reasonable and justifiable for inclusion in the model. Any model that has been modified must be viewed in an exploratory and hypothesis generating context, rather than as a confirmatory model. In this case, the paths added were of two types: 1) bi-directional relationships between climate variables and 2) direct paths from spatial variables to seed mass (Table S2, Fig. 2) [36]. Bidirectional relationships accounted for correlations between climate variables measured for the same site that were not explained by common latitude and longitude. Direct paths from spatial variables to seed mass were assumed to represent spatial effects that were not accounted for by climatic variables.

## Results

### Spatial variation in seed mass

Seed mass of black spruce and white spruce was negatively correlated with latitude, but the correlation was not significant in jack pine (Table 1, Fig. S2). This relationship was unaffected when the effect of altitude was controlled but changed when controlling longitude in partial correlation analyses due to the strong correlation between latitude and longitude of seed collection sites (Table S3). Seed mass was negatively correlated with longitude from east to west in black spruce and white spruce but positively correlated with longitude in jack pine regardless of the influence of latitude and altitude. Seed mass of black spruce was negatively correlated with altitude, but the correlations were not significant in white spruce and jack pine with or without controlling for latitude.

**Table 1:** Correlation coefficients (*r*) between seed mass and latitude, longitude and altitude across and within ecozones for black spruce, white spruce and jack pine in the Canadian Boreal Forest.

		n	Black spruce	n	White spruce	n	Jack pine
Across ecozones	Latitude	502	−0.30^***^	482	−0.10*	454	−0.04
	Latitude (control longitude)		−0.06		0.16^***^		−0.32^***^
	Latitude (control altitude)		−0.22^***^		−0.07		−0.05
	Latitude (control longitude and altitude)		−0.07		0.15^***^		−0.30^***^
	Longitude		−0.36^***^		−0.28^***^		0.18^***^
	Longitude (control latitude)		−0.21^***^		−0.31^***^		0.36^***^
	Longitude (control altitude)		−0.24^***^		−0.31^***^		0.23^***^
	Longitude (control latitude and altitude)		−0.13^***^		−0.34^***^		0.37^***^
	Altitude		−0.39^***^		−0.08		<0.001
	Altitude (control latitude)		−0.34^***^		−0.03		0.03
	Altitude (control longitude)		−0.30^***^		0.15^***^		−0.14^***^
	Altitude (control latitude and longitude)		−0.30^***^		0.15^***^		−0.09
Atlantic Maritime	Latitude	148	0.03	94	−0.03	97	0.21^**^
	Longitude		0.10		0.11		0.25^**^
	Altitude		−0.02		−0.03		0.25^**^
Boreal Shield	Latitude	320	−0.27^***^	274	0.08	272	−0.38^***^
	Longitude		−0.56^***^		−0.37^***^		−0.16^**^
	Altitude		−0.36^***^		−0.03		−0.30^***^

* *p*≤0.05, ^**^ *p*≤0.01, ^***^ *p*≤0.001.

Correlations across ecozones were evaluated with or without the effects of other spatial variables being controlled.

Within the Boreal Shield Ecozone, seed mass of black spruce and jack pine had negative correlations with latitude (from south to north) and altitude but seed mass of white spruce was not significantly correlated with either latitude or altitude (Table 1, Fig. S2). Seed mass of all three species decreased along the gradient of longitude from east to the west. Within the Atlantic Maritime, seed mass of black spruce and white spruce was not significantly correlated with latitude, longitude or altitude, while seed mass of jack pine had a positive correlation with latitude and altitude, respectively, and decreased from east to the west.

### Relationships between seed mass and climatic factors

Adequate SEM model fits were achieved for all species (black spruce: χ^2^_5_ = 9.12, *P* = 0.104; white spruce: χ^2^_9_ = 12.66, *P* = 0.179; jack pine: χ^2^_3_ = 4.98, *P* = 0.184). These models explained 33.6%, 14.3% and 28.3% of the variation in seed mass for black spruce, white spruce and jack pine, respectively (Fig. 2). Seed mass of all three species was correlated with longitude, while seed mass of black spruce and jack pine was positively correlated with latitude, and for jack pine it was negatively correlated with altitude.

**Figure 3:**
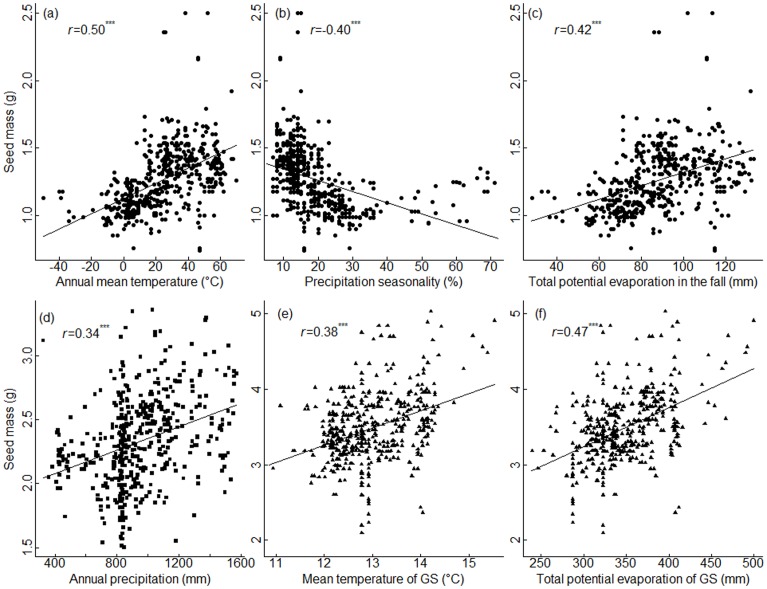
Relationships between seed mass and significant climatic variables in SEM. The climatic variables are annual mean temperature (a), precipitation seasonality (b), total potential evaporation in the fall (c) for black spruce (solid circle); annual precipitation (d) for white spruce (solid square); mean temperature of GS (growing season) (e) and total potential evaporation of GS (growing season) (f) for jack pine (solid triangle point-up).

Seed mass of black spruce increased with increasing annual mean temperature and total potential evaporation in the fall, and decreased with precipitation seasonality (Figs. 2, 3a, 3b, 3c). Seed mass of white spruce increased with annual precipitation and decreased with temperature seasonality (Figs. 2, 3d). For jack pine, seed mass increased with increasing mean temperature in the growing season, total annual precipitation and total potential evaporation in the growing season, but decreased with increasing temperature range of the growing season (Figs. 2, 3e, 3f).

## Discussion

### Geographical variation in seed mass

Seed mass is expected to decrease with increasing latitude and altitude [14], [16], [17], [20]. The negative correlation between seed mass and both latitude and altitude are consistent with ‘Baker's Rule’, which postulates that a reduced duration of growing season constrains the upper bound of seed mass [16], [18]. Small seeds at high latitude or altitude could be related to low temperature, low radiation, and short growing season [15], [16], [17]. The high solar radiation and long growing season at lower latitude or altitude can enhance the availability of photosynthate and increase reproductive allocation [15], [18], [20].This decline in seed mass with increasing latitude and altitude was not consistent among the three species in the present study, however. The partial correlation analyses demonstrate that longitude had a strong influence on the correlation between seed mass and latitude, likely due to the decline in average temperature and precipitation from coastal regions to the continental interior across the distribution range of the Canadian Boreal Forest. Within the Boreal Shield Ecozone precipitation increases from 1600 mm per year in the east to 400 mm per year in the west (http://canadianbiodiversity.mcgill.ca), and seed mass of black spruce and jack pine showed strong, negative correlations with both latitude and altitude but that of white spruce was not correlated with latitude and altitude. Within the Atlantic Maritime Ecozone, which is characterized by high precipitation and mild temperature (http://canadianbiodiversity.mcgill.ca), seed mass of black spruce and white spruce was not correlated with latitude and altitude while seed mass of jack pine was positively correlated with latitude and altitude. This suggests that environmental factors other than temperature and precipitation may become limiting.

Species-specific responses to climate were also reflected in correlations between seed mass and longitude. The seed mass of black spruce and white spruce decreased with longitude from east to the west but the trend was reversed in jack pine. The two spruce species have broader distribution within the boreal forest than jack pine (Table S3). Within the Boreal Shield Ecozone, seed mass of all the three species decreased with longitude from east to west, while seed mass of jack pine increased from east to west within the Atlanta Maritime Ecozone. Precipitation pattern is the main climatic variable contributing to the longitudinal variation in seed mass [13], [15].

### Species specific response of seed mass to climate

Climate, particularly temperature and precipitation, is the main environmental control in species distribution [39]. Geographic variation in seed mass is determined by that of climatic conditions [16]. Across the Canadian Boreal Forest, temperature and precipitation decrease with increasing latitude and longitude (from east to west). Results from the SEM demonstrated the regulation of seed mass by climatic factors and the species-specific adaptations in seed mass to long term climate.

Among climatic factors, annual mean temperature was identified as the main direct determinant of seed mass in black spruce, which takes effect via latitude and altitude. Annual precipitation, controlled primarily by longitude in the Canadian Boreal Forest as the distance to ocean increases from east to the continental interior, had the greatest influence on seed mass of white spruce. Favourable temperature and moisture conditions provide sufficient photosynthate for the development of large seeds [14], [40]. However, correlations between seed mass and monthly mean temperatures indicate that higher winter, but not summer, temperatures favour heavier seeds in both spruce species. Across the Canadian Boreal Forest, low winter temperatures commonly reduce reproductive growth [41]. Seed mass of jack pine was strongly correlated with growing season mean temperature, temperature seasonality, annual precipitation and growing season evapotranspiration. High solar radiation can also enhance photosynthesis and subsequently increase seed mass if moisture is not limited [16]. However, precipitation during fall and winter was negatively correlated with seed mass of jack pine.

### Seed mass adaptation to climate

Adaptation is genetically based and is usually studied through differential phenotypic expression of ecotypes grown under the same condition known as the common garden approach. However, plant materials such as whole plants (e.g., adaptation of *Picea abies* among altitudinal ecotypes [42]) or seeds (e.g., physiological traits of *Picea asperata* [43]) used in such studies have experienced fluctuation in weather and other environmental conditions before these studies, which have contributed to their phenotypic variation. For across continental studies on long-lived tree species with large amount of collections, common garden approach is simply not feasible. It is a common practice in large scale studies and meta-analysis (e.g., analysis of seed mass between vegetation types, [6]) to use data collected from different locations and time, which do not differentiate adaptation to long term climate and responses to short term fluctuations in weather. The SEM can analyze large amount of variables together and partition the amount of variance due to these factors, in our case, climatic variables.

Climatic and spatial variables explained 33.6%, 14.3% and 28.3% of the variation in seed mass for black spruce, white spruce and jack pine respectively (Fig. 2). Unexplained, remaining variation in seed mass may include the influences of weather and other environmental conditions. Weather conditions in both current and previous years can influence seed development, quality and quantity [21], [23], especially in species which take more than one year to complete the reproductive process. The lower variation explained for white spruce is likely due to the species' broad ecological niche, high plasticity and adaptation to a wide range of habitats and soil conditions [31]. Generally, species with phenotypic plasticity can be distributed over a wide range of environmental conditions [44], such as white spruce. Black spruce and jack pine, on the other hand, have more specialized habitat requirements and less plasticity than white spruce. Black spruce is stress-tolerant and most common in wet soils and bogs [20], [31], while Jack pine is drought-resistant and common in poor and sandy soils [21], [31]. Therefore, white spruce may cope better with changing climate than black spruce and jack pine.

Adaptation to local environmental conditions especially climate has survival values. Across the Canadian Boreal Forest climate varies from dry, cold continental in the interior to the wet, mild maritime in coasts with higher temperature and precipitation [31]. More favourable climates are associated with stronger competition [45], [46], conditions which gives larger seeds competitive advantage because they can produce bigger seedlings with greater survival rates and increased competitive ability [40]. The occurrence of damage caused by insect pathogens is also higher under warm and wet conditions [47]. Bigger seeds may have thicker seed coats to avoid insect and pathogen attacks and greater reserves to provide secondary compounds for defense against these attacks [5], [48]. Wildfire is more frequent in the interior and western regions due to their continental climate [49]. Other factors not addressed by the present study that may affect seed mass include soil nutrition and seed predation [16].

It is predicted that temperate tree species will expand their range northward and that suitable habitats at the southern end of their original range will be lost under climate change [50]. Disturbance regimes such as fire and disease may be modified by climate change. Fire is expected to be more frequent in the western regions and competition is expected to be greater in the eastern regions [47]. The reproduction and distribution of plant species may be modified by both direct effects of changes associated with temperature and precipitation, and indirect effects of changes in the disturbance regime in the Canadian Boreal Forest. Unlike weedy species whose seed traits enable them to evolve relatively quickly to keep pace with climate change [3], native species especially long living trees may not possess sufficient phenotypic plasticity and/or evolve fast enough to cope with climate change. Therefore, increased research on key issues related to the evolution and adaptation of seed traits is needed to fully comprehend and plan for plant response to global climate change [3].

## Conclusions

Spatial variation in seed mass of the three dominant tree species across the Canadian Boreal Forest was mainly determined by the longitudinal gradient with temperature and precipitation being the main climatic factors. Results of SEM showed that climatic variables explained a large portion of geographic variation in seed mass. Species with more specific habitat requirements and less plasticity such as black spruce and jack pine had more variation in seed mass explained by climate than more plastic species such as white spruce. Annual mean temperature and winter precipitation were positively correlated with seed mass of black spruce, but annual precipitation was the strongest correlate in white spruce. The combination of factors including growing season temperature and evapotranspiration, temperature seasonality and annual precipitation together determine seed mass of jack pine. Overall, sites with higher winter temperatures favour the production of large seeds. Species-specific adaptation to climate at the reproductive stage may lead to different shift in geographical distribution of tree species in response to climate change.

## Supporting Information

Figure S1 **Initial pathway models for seed mass for black spruce, white spruce and jack pine.** Variables are explained in the caption of Fig. 2.
(DOCX)

Figure S2 **Correlations in seed mass with latitude, longitude and altitude for black spruce (a, b, c), white spruce (d, e, f) and jack pine (g, h, i) across the Canadian Boreal Forest and within major ecozones.** Only significant correlations were presented. ^*^ *p*≤0.05, ^**^ *p*≤0.01, ^***^ *p*≤0.001.
(DOCX)

Table S1 Correlation coefficients (*r*) between seed mass and climatic variables for black spruce, white spruce and jack pine in the Canadian Boreal Forest.
(DOCX)

Table S2 Unstandardized path coefficients, standard error of the unstandardized coefficients, tests of path coefficient significance, and standardized path coefficients from the observed variable model in Fig. 2. Variable names are explained in the legend of Fig. 2. Standardized path coefficients are in standard deviation units and are primarily used to compare the relative strengths of paths within a given model. Unstandardized path coefficients represent the effect of a change in one variable on the other in absolute terms (equivalent to the slope of a regression model). For example, the unstandardized coefficient of −5.626 for the effect of latitude on annual temperature indicates that annual temperature decreased 0.5626°C with each one degree increase of latitude. The critical ratio (CR) is the unstandardized coefficient divided by its standard error. The CR statistic follows a t-distribution and is used to test the hypothesis of whether the unstandardized coefficient is significantly different from zero.
(DOCX)

Table S3 Correlation coefficients (*r*) between geographical variables for black spruce, white spruce and jack pine in the Canadian Boreal Forest.
(DOCX)
