# Supplementary material for: Patterns of Cross-Continental Variation in Tree Seed Mass in the Canadian Boreal Forest
Source: PLoS One. 2013 Apr 11;8(4):e61060. doi: 10.1371/journal.pone.0061060 (PMC3623855; doi:10.1371/journal.pone.0061060)
Supplement: Figure S1 — Initial pathway models for seed mass for black spruce, white spruce and jack pine. Variables are explained in the caption of Fig. 2. (DOCX) [file pone.0061060.s001.docx]

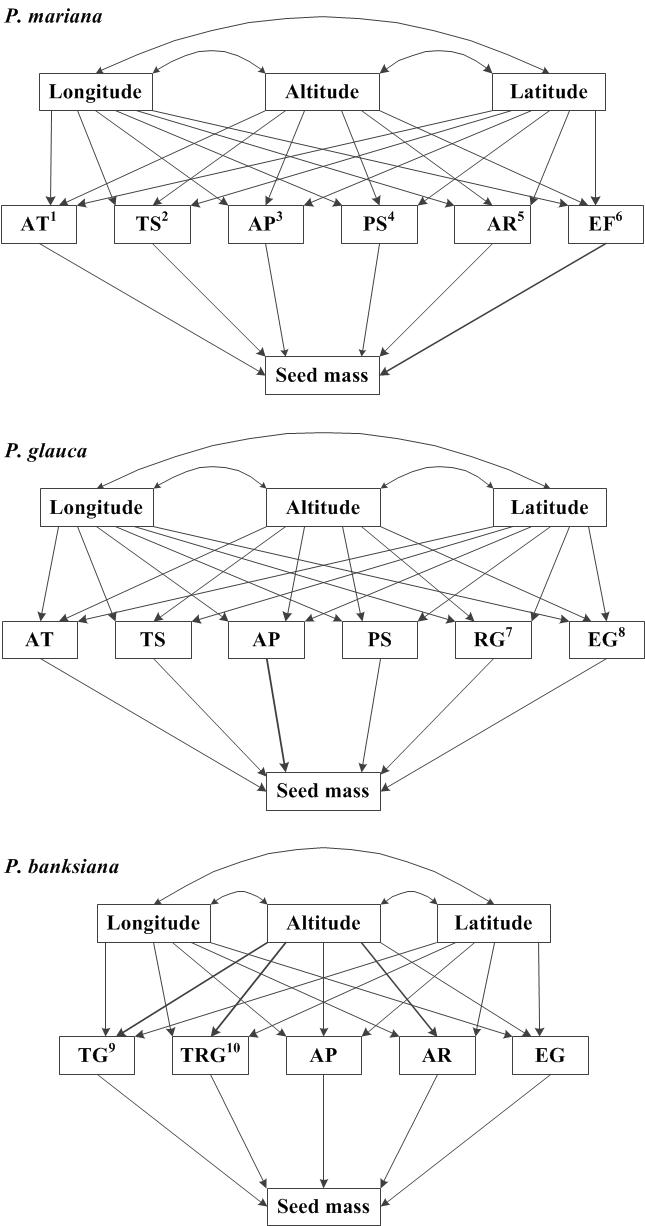


**Figure S1**. Initial pathway models for seed mass for black spruce, white spruce and jack pine. Variables are explained in the caption of Fig. 2.
