# Supplementary material for: Patterns of Cross-Continental Variation in Tree Seed Mass in the Canadian Boreal Forest
Source: PLoS One. 2013 Apr 11;8(4):e61060. doi: 10.1371/journal.pone.0061060 (PMC3623855; doi:10.1371/journal.pone.0061060)
Supplement: Figure S2 — Correlations in seed mass with latitude, longitude and altitude for black spruce (a, b, c), white spruce (d, e, f) and jack pine (g, h, i) across the Canadian Boreal Forest and within major ecozones. Only significant correlations were presented. * p≤0.05, ** p≤0.01, *** p≤0.001. (DOCX) [file pone.0061060.s002.docx]

**Figure S2**. Correlations in seed mass with latitude, longitude and altitude for black spruce (a, b, c), white spruce (d, e, f) and jack pine (g, h, i) across the Canadian Boreal Forest and within major ecozones. Only significant correlations were presented. ^*^ *p*≤0.05, ^**^ *p*≤0.01, ^***^ *p* ≤0.001.
