# Supplementary material for: Patterns of Cross-Continental Variation in Tree Seed Mass in the Canadian Boreal Forest
Source: PLoS One. 2013 Apr 11;8(4):e61060. doi: 10.1371/journal.pone.0061060 (PMC3623855; doi:10.1371/journal.pone.0061060)
Supplement: Table S1 — Correlation coefficients (r) between seed mass and climatic variables for black spruce, white spruce and jack pine in the Canadian Boreal Forest. (DOCX) [file pone.0061060.s003.docx]

**Table S1**. Correlation coefficients (*r*) between seed mass and climatic variables for black spruce, white spruce and jack pine in the Canadian Boreal Forest.

| Climatic variable | | Black spruce | White spruce | Jack pine |
| --- | --- | --- | --- | --- |
|  |  | 502 | 482 | 454 |
| Temperature | Annual mean temperature | 0.51^***^ | 0.06 | 0.16^***^ |
|  | Annual minimum temperature | 0.54^***^ | 0.11 | 0.11^*^ |
|  | Annual maximum temperature | 0.45^***^ | -0.01 | 0.23^***^ |
|  | Mean diurnal range^1^ | -0.40^***^ | -0.24^***^ | 0.18^***^ |
|  | Isothermality^2^ | 0.24^***^ | 0.02 | 0.22^***^ |
|  | Temperature seasonality^3^ | -0.54^***^ | -0.25^***^ | <0.001 |
|  | Temperature annual range^4^ | -0.52^***^ | -0.26^***^ | 0.03 |
|  | Mean temperature for period 3^5^ | 0.14^***^ | -0.18^***^ | 0.38^***^ |
|  | Temperature range for period 3 | 0.13^**^ | -0.20^***^ | 0.33^***^ |
|  | Max temperature of warmest period | 0.15^***^ | -0.19^***^ | 0.34^***^ |
|  | Min temperature of coldest period | 0.56^***^ | 0.19^***^ | 0.06 |
|  | Mean temperature of wettest quarter | -0.30^***^ | -0.24^***^ | 0.25^***^ |
|  | Mean temperature of driest quarter | 0.41^***^ | 0.24^***^ | -0.07 |
|  | Mean temperature of warmest quarter | 0.29^***^ | -0.12^**^ | 0.28^***^ |
|  | Mean temperature of coldest quarter | 0.56^***^ | 0.18^***^ | 0.08 |
|  | January mean maximum temperature | 0.54^***^ | 0.20^***^ | 0.07 |
|  | February mean maximum temperature | 0.53^***^ | 0.11^*^ | 0.16^***^ |
|  | March mean maximum temperature | 0.47^***^ | <0.001 | 0.25^***^ |
|  | April mean maximum temperature | 0.13^***^ | -0.24^***^ | 0.43^***^ |
|  | May mean maximum temperature | -0.08 | -0.26^***^ | 0.44^***^ |
|  | June mean maximum temperature | -0.07 | -0.23^***^ | 0.43^***^ |
|  | July mean maximum temperature | 0.12^***^ | -0.20^***^ | 0.35^***^ |
|  | August mean maximum temperature | 0.27^***^ | -0.17^***^ | 0.33^***^ |
|  | September mean maximum temperature | 0.40^***^ | -0.06 | 0.26^***^ |
|  | October mean maximum temperature | 0.45^***^ | 0.01 | 0.24^***^ |
|  | November mean maximum temperature | 0.49^***^ | 0.16^***^ | 0.06 |
|  | December mean maximum temperature | 0.54^***^ | 0.20^***^ | 0.04 |
|  | January mean minimum temperature | 0.56^***^ | 0.20^***^ | 0.06 |
|  | February mean minimum temperature | 0.57^***^ | 0.15^***^ | 0.12^**^ |
|  | March mean minimum temperature | 0.57^***^ | 0.11^*^ | 0.16^***^ |
|  | April mean minimum temperature | 0.51^***^ | 0.01 | 0.27^***^ |
|  | May mean minimum temperature | 0.28^***^ | -0.11^*^ | 0.30^***^ |
|  | June mean minimum temperature | 0.23^***^ | -0.12^**^ | 0.28^***^ |
|  | July mean minimum temperature | 0.39^***^ | -0.04 | 0.16^***^ |
|  | August mean minimum temperature | 0.43^***^ | 0.02 | 0.10^*^ |
|  | September mean minimum temperature | 0.42^***^ | 0.07 | 0.03 |
|  | October mean minimum temperature | 0.43^***^ | 0.11^*^ | <0.001 |
|  | November mean minimum temperature | 0.50^***^ | 0.18^***^ | -0.01 |
|  | December mean minimum temperature | 0.56^***^ | 0.21^***^ | 0.03 |
|  | Julian day number at end of the growing season | -0.21^***^ | 0.16^***^ | -0.38^***^ |
|  | Julian day number of start of the growing season | 0.42^***^ | 0.13^*^ | -0.04 |
|  | Number of days of the growing season | 0.40^***^ | 0.01 | 0.13^**^ |
|  | GDD above base temperature for period 1^6^ | -0.43^***^ | -0.13^**^ | -0.17^***^ |
|  | GDD above base temperature for period 2^7^ | -0.28^***^ | -0.19^***^ | 0.04 |
|  | GDD above base temperature for period 3 | 0.29^***^ | -0.12^*^ | 0.28^***^ |
|  | GDD above base temperature for period 4^8^ | 0.34^***^ | -0.10^*^ | 0.27^***^ |
| Precipitation | Annual precipitation | 0.40^***^ | 0.34^***^ | -0.14^**^ |
|  | Precipitation seasonality^9^ | -0.40^***^ | -0.18^***^ | 0.08 |
|  | Precipitation of wettest period | 0.11^***^ | 0.32^***^ | -0.18^***^ |
|  | Precipitation of driest period | 0.45^***^ | 0.28^***^ | -0.03 |
|  | Precipitation of wettest quarter | 0.24^***^ | 0.33^***^ | -0.18^***^ |
|  | Precipitation of driest quarter | 0.47^***^ | 0.33^***^ | -0.12^*^ |
|  | Precipitation of warmest quarter | 0.14^***^ | 0.32^***^ | -0.06 |
|  | Precipitation of coldest quarter | 0.45^***^ | 0.33^***^ | -0.14^*^ |
|  | January total precipitation | 0.46^***^ | 0.34^***^ | -0.16^***^ |
|  | February total precipitation | 0.45^***^ | 0.33^***^ | -0.12^*^ |
|  | March total precipitation | 0.46^***^ | 0.30^***^ | -0.10^*^ |
|  | April total precipitation | 0.42^***^ | 0.27^***^ | -0.05 |
|  | May total precipitation | 0.37^***^ | 0.30^***^ | -0.07 |
|  | June total precipitation | 0.23^***^ | 0.28^***^ | 0.02 |
|  | July total precipitation | -0.05 | 0.29^***^ | -0.12^**^ |
|  | August total precipitation | 0.21^***^ | 0.31^***^ | -0.01 |
|  | September total precipitation | 0.09^*^ | 0.30^***^ | -0.23^***^ |
|  | October total precipitation | 0.36^***^ | 0.31^***^ | -0.18^***^ |
|  | November total precipitation | 0.40^***^ | 0.32^***^ | -0.15^**^ |
|  | December total precipitation | 0.44^***^ | 0.32^***^ | -0.15^**^ |
|  | Total precipitation for period 1 | 0.44^***^ | 0.30^***^ | -0.12^*^ |
|  | Total precipitation for period 2 | 0.31^***^ | 0.32^***^ | -0.15^***^ |
|  | Total precipitation for period 3 | 0.38^***^ | 0.28^***^ | -0.08 |
|  | Total precipitation for period 4 | 0.38^***^ | 0.27^***^ | -0.06 |
| Potential evaporation | Annual potential evaporation | 0.15^***^ | -0.21^***^ | 0.45^***^ |
|  | January potential evaporation | 0.05 | <0.001 | 0.02 |
|  | February potential evaporation | 0.04 | <0.001 | 0.04 |
|  | March potential evaporation | 0.27^***^ | 0.02 | 0.12^*^ |
|  | April potential evaporation | 0.22^***^ | -0.19^***^ | 0.43^***^ |
|  | May potential evaporation | <0.001 | -0.26^***^ | 0.46^***^ |
|  | June potential evaporation | -0.21^***^ | -0.27^***^ | 0.45^***^ |
|  | July potential evaporation | -0.05 | -0.27^***^ | 0.42^***^ |
|  | August potential evaporation | 0.12^**^ | -0.25^***^ | 0.45^***^ |
|  | September potential evaporation | 0.32^***^ | -0.11^*^ | 0.40^***^ |
|  | October potential evaporation | 0.41^***^ | -0.01 | 0.26^***^ |
|  | November potential evaporation | 0.48^***^ | 0.15^***^ | 0.06 |
|  | December potential evaporation | 0.19^***^ | 0.10^*^ | -0.09^*^ |
| Radiation | Annual radiation | 0.32^***^ | -0.13^**^ | 0.25^***^ |
|  | January radiation | 0.32^***^ | -0.01 | 0.14^**^ |
|  | February radiation | 0.27^***^ | -0.03 | 0.15^***^ |
|  | March radiation | 0.20^***^ | -0.10^*^ | 0.18^***^ |
|  | April radiation | 0.06 | -0.21^***^ | 0.25^***^ |
|  | May radiation | 0.09^*^ | -0.18^***^ | 0.16^***^ |
|  | June radiation | 0.25^***^ | -0.18^***^ | 0.31^***^ |
|  | July radiation | 0.17^***^ | -0.22^***^ | 0.31^***^ |
|  | August radiation | 0.30^***^ | -0.16^***^ | 0.25^***^ |
|  | September radiation | 0.43^***^ | -0.07 | 0.25^***^ |
|  | October radiation | 0.41^***^ | -0.06 | 0.26^***^ |
|  | November radiation | 0.38^***^ | -0.05 | 0.17^***^ |
|  | December radiation | 0.35^***^ | -0.02 | 0.16^***^ |

^*^ *p*≤0.05, ^**^ *p*≤0.01, ^***^ *p* ≤0.001.

^1^Mean diurnal range: mean (month max-min).

^2^Isothermality: mean diurnal range/temperature annual range.

^3^Temperature seasonality: the standard deviation of the monthly mean temperatures expressed as a percentage of the mean of those temperatures (i.e. the annual mean).

^4^Temperature annual range: max temperature of warmest month - min temperature of coldest month.

^5^ Period 3: growing season.

^6^Period 1: 3 months prior to the start of growing season.

^7^ Period 2: the 1st six weeks of growing season.

^8^ Period 4: difference between period 3 and period 2.

^9^Precipitation seasonality: standard deviation of monthly precipitation estimates expressed as a percentage of the mean of those estimates (i.e. the annual mean).
