# Supplementary material for: Patterns of Cross-Continental Variation in Tree Seed Mass in the Canadian Boreal Forest
Source: PLoS One. 2013 Apr 11;8(4):e61060. doi: 10.1371/journal.pone.0061060 (PMC3623855; doi:10.1371/journal.pone.0061060)
Supplement: Table S2 — Unstandardized path coefficients, standard error of the unstandardized coefficients, tests of path coefficient significance, and standardized path coefficients from the observed variable model in Fig. 2. Variable names are explained in the legend of Fig. 2. Standardized path coefficients are in standard deviation units and are primarily used to compare the relative strengths of paths within a given model. Unstandardized path coefficients represent the effect of a change in one variable on the other in absolute terms (equivalent to the slope of a regression model). For example, the unstandardized coefficient of −5.626 for the effect of latitude on annual temperature indicates that annual temperature decreased 0.5626°C with each one degree increase of latitude. The critical ratio (CR) is the unstandardized coefficient divided by its standard error. The CR statistic follows a t-distribution and is used to test the hypothesis of whether the unstandardized coefficient is significantly different from zero. (DOCX) [file pone.0061060.s004.docx]

**Table S3**. Correlation coefficients (*r*) between geographical variables for black spruce, white spruce and jack pine in the Canadian Boreal Forest.

|  |  | Black spruce | |
| --- | --- | --- | --- |
|  |  | Latitude | Longitude |
| Black spruce | Longitude | 0.73^***^ |  |
|  | Altitude | 0.27^***^ | 0.39^***^ |
| White spruce | Longitude | 0.73^***^ |  |
|  | Altitude | 0.51^***^ | 0.68^***^ |
| Jack pine | Longitude | 0.81^***^ |  |
|  | Altitude | 0.58^***^ | 0.61^***^ |

^*^ *p*≤0.05, ^**^ *p*≤0.01, ^***^ *p* ≤0.001.
